# How to Treat Depression With Low-Intensity Virtual Reality Interventions: Perspectives on Translating Cognitive Behavioral Techniques Into the Virtual Reality Modality and How to Make Anti-Depressive Use of Virtual Reality–Unique Experiences

**DOI:** 10.3389/fpsyt.2019.00792

**Published:** 2019-10-31

**Authors:** Philip Lindner, William Hamilton, Alexander Miloff, Per Carlbring

**Affiliations:** ^1^Department of Psychology, Stockholm University, Stockholm, Sweden; ^2^Centre for Psychiatry Research, Department of Clinical Neuroscience, Karolinska Institutet, & Stockholm Health Care Services, Stockholm County Council, Stockholm, Sweden; ^3^Center for Dependency Disorders, Stockholm Health Care Services, Stockholm County Council, Stockholm, Sweden; ^4^Mimerse, Stockholm, Sweden; ^5^Department of Psychology, University of Southern Denmark, Odense, Denmark

**Keywords:** depression, virtual reality, cognitive behavior theory, human–computer interaction, self-help

## Abstract

Depression is a common mental disorder with a large treatment gap. Low-intensity, automated virtual reality (VR) interventions (not requiring a therapist) is a scalable and promising solution now that VR is an accessible and mature, consumer technology. Yet unlike with phobias, there have been few attempts at translating evidence-based cognitive behavioral therapeutic (CBT) techniques for depression into the VR modality. In this paper, we discuss how specific CBT techniques can be made into VR experiences, including psychoeducation, behavioral activation, cognitive restructuring, and social skills training. We also discuss how VR-unique experiences, such as alternative embodiment and virtual pet interactions, can be made therapeutic. Creating a pre-clinical and clinical evidence base for these types of novel interventions should be considered a research priority, and high-quality development on par with other consumer VR applications will be essential to the success of any consumer-targeted intervention. If this is achieved, low-intensity VR interventions for depression have great potential to make an impact on public mental health.

## Introduction

Depression is a major contributor to the global burden of disease (1), with an estimated 10% of the Swedish population reporting clinically significant symptoms (2). While spontaneous remission is not uncommon (3), depression episodes commonly become recurrent (4) and only an estimated half of sufferers seek treatment (5). Cognitive behavior therapy (CBT) refers to a diverse yet coherent collection of therapeutic techniques that aim to promote change in behavior and cognition. CBT is demonstrably effective in reducing depressive symptoms (6) and has proven remarkably flexible in terms of format: in addition to the traditional therapist-led format, Internet-delivered CBT self-help, with or without guidance, is also an evidence-based intervention for depression (7). Burgeoning evidence suggests that smartphone applications are also a viable and efficacious delivery format (8).

Another consumer technology with potential to act as a delivery format for CBT for depression is Virtual Reality (VR), a technology that allows the user to feel immersed in virtual, computer-generated world (9). This is typically achieved using a head-mounted display that withholds the outside world, while a stereoscopic display creates the illusion of depth perception, the presentation of which is interactive to head movements (measured using gyroscopes), allowing the user to look around the virtual environment. Sound effects can be used to strengthen immersion, and handheld controllers provide an additional means of interacting with the virtual environment by acting as a mouse pointer or virtual hands. High-quality, standalone mobile VR platforms now cost as little as 240 USD and feature mature and rich digital marketplaces for easy dissemination of applications, making VR an affordable and accessible consumer technology (10).

VR technology provides full control over the user’s experience, which can be used for therapeutic purposes. Most past research on VR interventions for mental health have been focused on treating anxiety disorders (9, 11), and for pain distraction (12, 13) and relaxation (14, 15). There is ample evidence to support efficacy in these areas, even as automated self-help applications (16–18). There is however a striking dearth of VR interventions for depression (19). From a technological perspective, it is relatively easy to present the user with either a virtual equivalent of the phobic stimulus to enable exposure therapy (for anxiety disorders), use immersive game mechanics for sensory distraction (for pain relief), or situating the user in a calming natural environment (for relaxation). As evident by the scarce extant literature, translating evidence-based CBT techniques used in treating depression into the VR modality has however proven a much greater challenge.

In this paper, we discuss ways of translating traditional CBT techniques for depression into the VR modality, as well as how to make use of the inherent capabilities of VR-unique experiences to treat depression, especially in the form of low-intensity interventions. We hope that these discussion points inspire and inform a new generation of consumer-targeted VR interventions for depression.

## Translating CBT Techniques Into VR

### Immersive Psychoeducation and Problem Solving

Almost all psychotherapeutic techniques require psychoeducation to some degree, which at minimum includes conveying a coherent conceptualization of the symptom/syndrome in question, and possibly a derived treatment rationale and instructions for exercises. Even passive psychoeducation has a small anti-depressive effect (20). In CBT, this often includes generic problem-solving skills such as breaking larger goals and tasks into smaller units, how to plan, record and evaluate exercises, brainstorming ideas and filtering, and similar skills.

Immersive VR experiences rely on a clear, binary distinction between the virtual and real world, making it suboptimal to use VR as a bridge between the therapeutic context (planning, tracking, and evaluating) and the real-life setting where the exercise is performed. Traditional pen-and-paper, or companion smartphone applications are better choices here. VR is however inherently very well suited for passive psychoeducation. Recent automated VR applications have taken conservative approaches to conveying psychoeducation in VR, e.g., in the form of a virtual therapist office and projector screen (18), yet VR can be used to create much more immersive and thereby likely efficacious psychoeducation. Here, the fields of educational and entertainment VR may serve as an inspiration and source of knowledge. VR is considered a powerful educational tool since it allows user to *experience*, rather than simply *perceive*, what is to be learned (21). Thus, VR holds an advantage over traditional, non-immersive delivery only when the experience is complex, dynamic, exciting, and instills a sense of presence (22, 23). CBT often encourages viewing things as objectively as possible in order to disprove experiences and cognitions that are contaminated by negative emotions. Using VR, the user could for example first experience a learning example scenario in the first or second person, and then view the same scenario as a third person (observer) and be asked to note discrepancies. Further, with VR, it is not only possible but preferable to animate the therapeutic material to a coherent and continuous narrative, pausing only for interactive elements that can be used to tailor the experience. A recently developed, single-session VR intervention for depressed adolescents that teaches and encourages a growth mindset, incorporates many of these elements (24).

### Behavioral Activation and Physical Activity

Behavioral activation (BA) is a core part of CBT for depression and departs from an operant view of depression as a vicious circle of withdrawal from rewarding activities (25), the remedy for which is functional assessment and stepwise activity scheduling in order for the patient to once again come in contact with natural reinforcers (26). While it would certainly be appropriate to present the treatment rational and specific techniques in an immersive fashion using VR (as discussed above), actually performing appropriate activities in VR has some inherent caveats. Primarily, BA typically focuses on decreasing solitary, at-home technology use (often part of the clinical presentation of depression) and instead promoting engagement with the outside world. Simply recreating VR equivalents of the everyday tasks often used as targets in ordinary BA is thus unlikely to be effective or appropriate, although this is ultimately an empirical question, as of yet unanswered.

Physical activity and social gatherings in VR would however be clinically appropriate targets to increase the frequency of. From a behavioral therapeutic perspective, physical activity can be seen as a special case of BA, and there are several large trials showing comparable effects on depression of BA versus physical activity (27, 28). Combining VR and moderate physical activity is relatively straightforward: any type of physical activity that can be performed on the spot and involves no quick, continuous head movements (which would induce motion sickness) can be done while wearing a mobile VR unit. In fact, VR exercise applications are some of the most popular on digital marketplaces, offering activities, such as VR boxing and tennis. Combining such activities with a BA treatment rationale (27) within a dedicated application has the potential to achieve similar anti-depressive effects as traditional physical activity.

The other appropriate VR BA target is to engage in virtual social gatherings. There are already several popular social VR applications that allow users not only to meet through avatars and voice chat but also to share experiences such as playing board games, singing karaoke, hosting movie nights, attending concerts, and more. A dedicated VR BA application could be designed to suggest social activities based on provided data and direct users to virtual gatherings through linkage to existing social VR applications.

### Cognitive Restructuring

**Figure 1:**
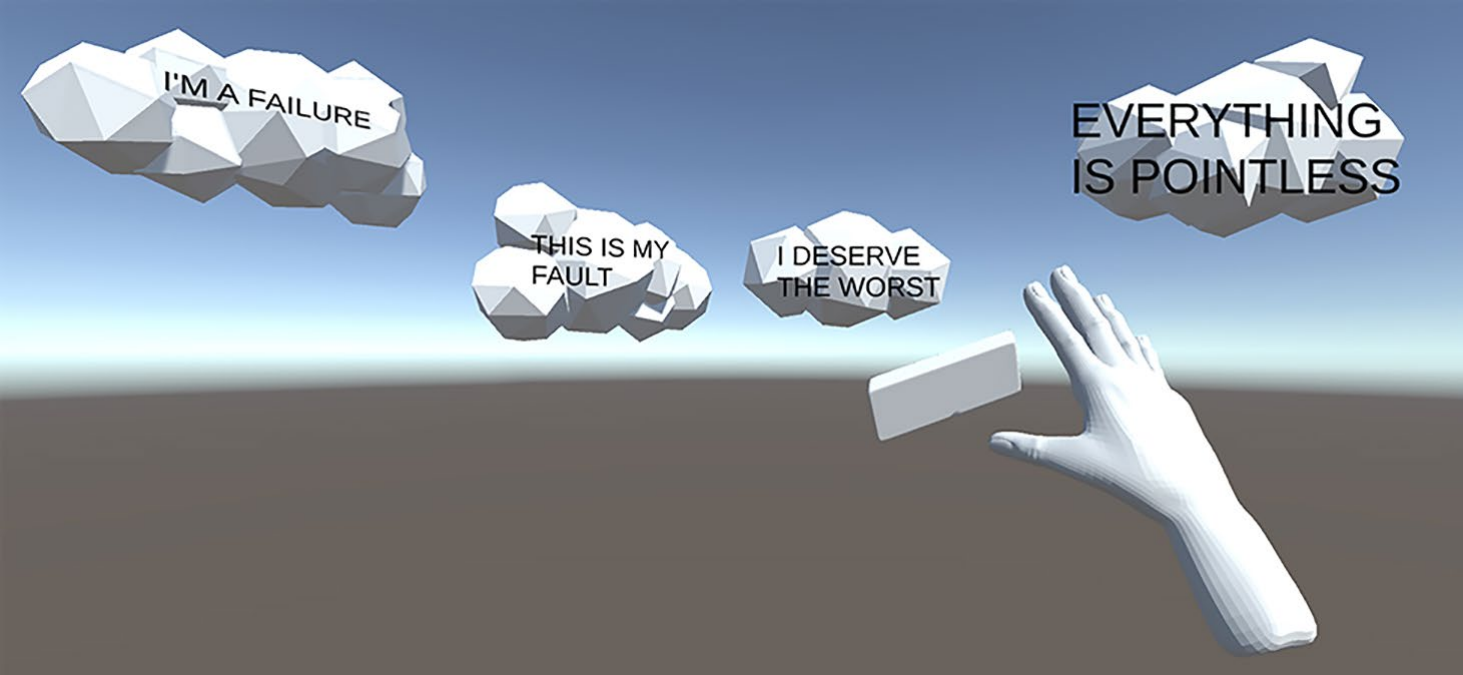
Mockup of using VR for hands-on cognitive restructuring.

Cognitive restructuring refers to a collection of therapeutic techniques designed to identify cognitive patterns that maintain depressed mood, and to modify these (29). We see two attractive alternatives for translating this set of techniques into the VR modality: using VR as interactive visualization aid, and for imagery rescripting and positive imagery training. The former would entail making use of the powerful visual capabilities of VR and having the user create, manipulate, and observe therapeutic material in an immersive manner. Many cognitive exercises require the patient to imagine an example situation from which to extract and manipulate cognitions for exercise purposes; VR would enable virtually situating the user in this example situation, playing the example scenario, allowing the user to pause the scenario, and e.g. report negative automatic thoughts by visualizing them as speech balloon type objects. Handheld controllers may then be used to manipulate these objects in accordance with the aim of the exercise, e.g., using a virtual eraser, drag-and-drop sorting of matching cognitive distortions, or identifying evidence for and against underlying assumptions, etc. See **Figure 1** for a demonstration. One recent study (30) provides a proof-of-concept for this approach, allowing the user to suspend self-critical words in virtual space and manipulate these objects with handheld controllers. Lasting clinical efficacy on depressive symptoms has however not yet been demonstrated.

A related type of cognitive exercise well-suited for the VR modality is imagery rescripting (31, 32) and positive imagery training (33). Both these techniques involve the patient using mental imagery to either recreate a memory (rescripting) or expand upon an example scenario in a prompted direction (positive imagery training), in their mind, as vividly as possible. In the case of rescripting, the patient is tasked with finding alternative solutions to the imagined situation that reduces their distress, and then rehearses a novel solution in greater and greater detail. In positive imagery training, the patient is instead prompted with a (positive) message and tasked with imagining the outcome, and repeating with a new scenario and message. In both these techniques, imagery vividness has been postulated as an important mediator of treatment effects, as is the case with imaginal exposure for anxiety disorders (34). Alas, there is great variability between individuals in vividness of mental imagery, measured both subjectively and with neuroimaging (35). VR could alleviate this issue by either completely replacing mental imagery with actual virtual scene-building and experiencing different alterations, or by acting as a powerful visual booster prior to subsequent mental imagery in order to increase vividness without the need for full scene-building.

An application for the latter would be easier to develop yet would still require mental imagery and be susceptible to inter-individual differences in vividness, while the former would require an advanced and content-rich sandbox type application (non-VR equivalents of which are already commercially available). Perfect agreement between the virtual scene and a memory is unlikely to ever be achieved, yet it is an open empirical question whether this is necessary or beneficial. Decades of research on VR exposure therapy for post-traumatic stress disorder with good but non-perfect agreement (36) suggests that this is not a prerequisite for efficacy. Congruently, one recent, innovative but low-powered study on exposure therapy for worry compared standardized VR scenarios against personalized imaginal exposure and found no difference in elicited anxiety (37). These findings do not however rule out the possibility that greater effects are possible with better agreement between idiosyncratic imagery and the VR material presented.

### Social Skills Training

Social skills training is a generic CBT technique that conceptualizes everyday social behaviors as skills to be taught, practiced, and implemented in order to live a functional and fulfilling life (38). Such skills may include correctly interpreting and norm-appropriate responding to verbal and non-verbal social cues, conversational skills, assertiveness training, and relationship building. VR is uniquely well suited for training social skills *via* virtual conversational agents and immersive scenarios. VR social skill training interventions for autism (39) and schizophrenia (40) have already been evaluated and show promise, and the generic nature of this therapeutic technique suggests equally good results for depression.

## Making Anti-Depressive Use of VR-Unique Experiences

### Embodiment Experiences

VR allows unique and powerful experiences, which can be translated into unconventional clinical applications, granted that there is a theoretical rationale and pre-clinical evidence to support efficacy. One innovative example used VR and virtual embodiment to address self-criticism in depressed patients by having them first convey compassion to a crying virtual child while embodied in a virtual adult body, then receive the same, motion- and audio-captured acts of compassion when embodied in the child’s body, leading to decreased self-criticism and depressive symptoms (41). So-called avatar therapy is a related concept that could be made more powerful with VR (42) and possibly used in treating depression. Originally developed for patients with psychosis, avatar therapy involves the patient creating a virtual avatar and externalizing auditory verbal hallucinations to this avatar through seeing and hearing this avatar perform lines that the patient would otherwise have attributed to the verbal hallucinations (43). Avatar therapy could, in principle, also be used to externalize the self-criticism and other negative automatic thoughts frequently reported by depressed patients. This may help make cognitive exercises in identifying and disproving them, more concrete and easier to execute, leading to decreased frequency and alleviated depressive symptoms.

### Positive Affect Through Virtual Gardens and Animals

The majority of CBT techniques target the excess of negative affect (sadness) aspect of depression, and even techniques such as BA have a limited effect on the deficit in positive affect (anhedonia) (44). A recently developed CBT paradigm, which includes augmented BA, mental imagery, and specifically targets deficit reward sensitivity, has however been shown to reduce anhedonia (45). Performing specific types of pleasurable activities in VR, and using virtual perspective changing and alternative embodiment in lieu of mental imagery, is both feasible and clinically appropriate; a clinical trial using a related approach is ongoing (NCT03715400). Such pleasurable activities may include virtual gardening and virtual pet interactions, akin to concentrated forms of gardening therapy (46) and animal-assisted therapy (47).

## Discussion

Despite decades of research showing that VR technology can be used to treat phobias, stress and pain—which all require relatively simple virtual environments—there have been very few attempts to develop anti-depressive VR interventions. In this paper, we have discussed ways of translating existing CBT techniques into the VR modality, and how to make anti-depressive use of VR-unique experiences. While not without its challenges, we argue that some CBT techniques are not only suitable for the VR modality, but that the inherent capabilities of VR can even be used to augment the effect of these techniques.

Since VR is built on excluding the outside world and generating a virtual one, this medium is inherently well suited for delivery of so called low-intensity interventions that are scalable and require few resources to disseminate, which does not necessarily equate to being less intense for the person engaging with it. All the techniques discussed above can be automated, most at little extra development costs; interventions that require complex decision tress (e.g. to realistically mimic social interactions) will require more development resources but are certainly feasible using today’s technology. Automated VR treatments—for which there is growing evidence in the field of anxiety (16–18)—would then require no therapist to deliver and could be disseminated using ordinary digital marketplaces (18, 48) and reach hundreds of thousands of sufferers, promising a potent public health impact.

Unlike VR interventions for phobias, pain, and stress, for which there is already an evidence base, the field of anti-depressive VR applications is at an early stage, constituting an important limitation to the current state of research. Both pre-clinical research (e.g. on presumed mechanisms and human–computer interaction) and high-quality clinical trials should be considered a research priority. This is especially important for the VR-unique interventions discussed above, like alternative embodiment, where there is no evidence base from in-vivo equivalents. The field should strive to avoid a situation akin to the questionable clinical utility of consumer-targeted CBT smartphone applications for depression (49): however, the uniqueness of the VR medium along with the lack of an evidence base, entails that the future assortment of consumer-targeted VR interventions for depression available at digital marketplaces will likely end up with an equally poor clinical utility. Research must keep up with the expected market-driven application development in order to guide it. As with all low-intensity interventions and the stepped care model, inefficacious low-intensity VR interventions, or a mismatch between severity and care step, will delay care provided at the appropriate and satisfactory level.

All the VR techniques discussed above will benefit immensely from high-quality development on par with the commercial VR applications (e.g. regular games) that will realistically compete for the user’s time, especially if disseminated as consumer-targeted applications on regular digital marketplaces. High-quality development includes a visually pleasing graphical user interface, user-friendly design, gamification elements to promote repeated use, and other aspects that consumers expect from any application. In many VR therapeutic techniques, sense of presence not only moderates but also mediates outcomes, and presence-breaking elements and events must be avoided at all costs (including sacrificing maximum graphical fidelity). Careful design considerations should be made at an early stage in development and revised through iterative testing, taking into account core characteristics of both the disorder, therapeutic mechanism, and user experience (50).

## Conclusions

VR is a clinically appropriate treatment modality for many existing CBT techniques and the extant literature suggests that VR-unique experiences can be put to anti-depressive use. Low-intensity, consumer-targeted VR interventions built around evidence-based therapeutic techniques have great potential to decrease the depression treatment gap and make an impact on public mental health.

## Author Contributions

Drafted the manuscript: PL. Made significant contributions to the manuscript: WH, AM, and PC.

## Conflict of Interest

Author WH is the founder and chief technology officer of Mimerse, a company specializing in developing VR interventions for mental health. Author PL has consulted for Mimerse but holds no financial stake in the company. 
